# Notes of an European Tour

**Published:** 1845-11-01

**Authors:** F. H. Hamilton

**Affiliations:** Professor of Surgery in Geneva Medical College


### BUFFALO MEDICAL JOURNAL

Vol. 1. NOVEMBER 1, 1845. No. 6.

### Notes of an European Tour.---No. 6.

Communicated for the Buffalo Medical Journal, by F. H. HAMILTON, M. D., Professor of Surgery in Geneva Medical College.

DEAR M. SWITZERLAND.

We left Villeneuve at early day-break: and travelling on through Gex and several smaller towns, belonging still to the Canton Vaud, we reached the frontier of the Valais Canton at about 8 A. M. Here the Rhone rushes through a deep chasm formed apparently by a rending asunder of that long and lofty chain of mountains which traverses Switzerland nearly east and west. The bridge, an ancient stone structure, built by the Romans, rests at one extremity against the base of Dent de Morcles, and at the other against the perpendicular sides of Dent du Midi; it is two hundred feet in length and of prodigious height, guarded at one end by a stone tower and at the opposite by a half ruined castle. Crossing this bridge I entered "where a key unlocks a kingdom." St. Maurice, the first of the Valais towns, extends from the bridge close between the mountain and the river, being little more than a single narrow street. Here we were detained nearly three hours, waiting for the Savoy line of coaches.

The Valais Canton commencing at this "barriere" reaches upward along the Rhone towards its source; and from hence, the whole physical features of the country become suddenly changed. Hitherto the valley had been wide and but slightly shaded by its opposite mountains; from St. Maurice it is generally narrow, deep, and overhung by some of the loftiest Alps: until now the soil of the valley had been wet but fertile, hereafter it is cold, rocky, and sterile: the villages we had passed were neat and flourishing, those we are now entering, are dirty and miserable in the extreme. But it is in the inhabitants that the most striking contrast is seen. Here live a race of beings of low, dwarfish stature, with idiotic heads and tallowy complexion, diseased, deformed, and bearing always marks of premature old age, two thirds of whom carry enormous goitres and thousands of whom have scarcely more intelligence than the swine with which they kennel, eat and die: the same now as when Juvenal wrote "quis tumidum guttur miratur in Alpibus."

In this disgusting picture St. Maurice occupies the foreground, being situated at the narrowest and deepest part of the Valley, where also from the direction of the valley north and south, the sun has the least admission, so that the inhabitants vegetate like plants in a cellar and seem filled with a cold, watery, stagnant fluid

A cup of "Cafe aut lait" at the dirty Post House, was all my stomach would receive, and taking with me a ragged, toad-looking guide, who with that kind of instinct which belongs to the dullest of his profession, had marked me from my companions as a foreigner, I sauntered out on a tour of observation. Nearly opposite the "Post" was a fountain around which were gathered some dozen or more women, who with their prayer books were just returning from matin service; (this Canton you know is Catholic—the doctrines of Luther never crossed the bridge of St. Maurice,) not one of whom, I was impudent enough to ascertain, lacked the "tumid neck." Near the bridge I noticed several buildings or rather caverns forming the upper side of the street, built against and partly hewn from the perpendicular face of the rock, closed in front with a wall of stone and a rough wooden door, which was reached generally by a broken and steep stairway of ten or twelve steps. One of these I was proposing to enter and had already ascended more than half the steps, when two of the ugliest looking imps I ever saw began to assail me from the door with dirt and pieces of stone, accompanied with the most unnatural cries. It was evident that the place could only be taken by storm, and as I had no thought of any thing but a peaceable entrance I immediately beat a retreat. My villainous guide who stood grinning below at my discomfiture, now pointed to another "hole" a little beyond where he intimated that I might be admitted if I had a batzen to spare; the terms were reasonable and I accepted. A similar flight of stairs led to an open door and we entered without ceremony. It was a small, single room, one side of which was formed by the moist, black rock; the door served the triple purpose of place of entrance, chimney and window. A few extinguished embers near the back wall indicated the fire place. The furniture consisted of a box of straw for a bed, a table, bench, stools, with sundry pieces of broken iron which might have been called cooking utensils. The occupants of this place were a squabby, stupid looking woman, and an infant nearly naked, and which looked more like a young kangaroo than human offspring. As we entered, a few words passed between my guide and the woman, which ended in a half idiotic expression of pleasure on the face of the latter and an extension of her filthy hand for alms. I saw how much it had cost to sustain such an establishment—intellect, health, and every thing that can render life desirable—and I did not deny the claim.

Leaving St. Maurice we passed on the right "the Hermitage," a small chapel placed on a shelf half way up a perpendicular rock and accessible only by a narrow and difficult stairway which winds down from above.—The occupant, I was told, is a solitary monk. Beyond the Hermitage, near the road, is a small plain, marked by tradition as the spot where the Theban legion of six thousand soldiers refusing to abjure Christianity, was executed A. D. 302, by order of the Emperor Maximian. Farther on, the road becomes irregular, presenting traces of an avalanche of earth which a few years since slid from the summit of Dent du Midi, covering the road, orchards, fields and dwellings; granite blocks weighing many tons were carried down with such force as to bury themselves deep in the earth below.

Martigny is the next town and has little to distinguish it from its neighbor, either in its general aspect or the appearance of its population.—The ruined tower of La Batie, formerly the palace and castle of the Archbishops of Sion, is situated upon a high hill which overlooks the town from the west. Within the town is the large convent of St. Augustine Monks, from which the Hospices of St. Bernard and the Simplon are regularly relieved. Here also commences the St. Bernard track across the Alps, rendered famous by the passage of Napoleon. Leaving to Napoleon all the glory of this difficult pass, I chose to cross at the "Simplon" which leaves the valley about 50 miles farther up.

For a few miles after leaving Martigny, we drove along the left bank of the Rhone, (which here flows again toward the west) and through a scene of almost complete desolation. At Riddes we again took the right bank, and a mile farther on crossed the Licerne, a small tributary which descends from Mt. Diablerets nearly ten thousand feet high, and along whose southern slope the track of an avalanche is still visible. In 1714 a slide of unusual extent from this mountain crushed many cottages with their inhabitants; one person however escaped after being buried three months. His hamlet, built of stone, had not broken in, and supplied with cheese which he had provided for winter, and water which trickled through the walls, he at length contrived to work his way out.

All the way along the road I saw stupid beings, dragging themselves to their work and loitering in the fields, who as we passed would stop and gaze always with the same unmeaning stare. The cattle even looked rickety and the horses half fed—vegetation also partook of the same sickly appearance; the grass grew wet and rank, and the few trees of apple and pears which I saw were gnarled and dwarfish.

Looking up the valley, the city of Sion is seen situated in its centre, which with its two castles crowning a couple of abrupteminences near the middle of the town, presents as seen from a distance a highly picturesque appearance. As we approached I was surprised at the appearance of a military encampment just without the walls of the city, the whole plain adjoining the road being covered with tents and soldiers: and we entered under the gateway of the ancient Capital of the Seduni, now the See of a Catholic Bishop and Capital of the Valais Canton, amid a gay scene of floating banners and waving plumes. I had learned at Geneva that considerable popular excitement existed along this Valley in consequence of the general Diet having exercised certain authorities, by this Canton and others deemed unconstitutional; but the immediate occasion of the appearance of the military under the walls of Sion was that in obedience to a call circulated a few weeks before a "convocation extraordinaire du Canton du Haut Valais" had to-day met at the "Hotel de Ville" of Sion. This was considered illegal, and the troops were assembled by order of the Diet either to disperse or intimidate the assembly, or perhaps, in case of necessity to storm the city.

This ancient city, for several centuries a fortified ecclesiastical residence, one of whose Bishops had impressed upon his coin the effigy of a Devil, I will describe to you by quoting verbatim my notes made when after a short walk I returned to my Hotel. "Walls broken; streets narrow, crooked, damp, filthy; crammed with soldiers, priests, peasants, goats, jacks, donkeys, cats, dogs and parrots; men dwarfish, deformed, blear-eyed, black and devilish; women ill-formed, squabby, goitrous, yellow, ugly, wizard looking, with dirty valais hats or wash tubs on their heads; met a priest with a shorn scalp, bareheaded, barefooted, long beard, greasy frock and cowl, rope about the waist, face bespattered with snuff, with a tin box, and pewter cross, begging alms to pay himself for praying poor souls out of purgatory. (If I am forced to stay long here I shall ask for his prayers.) Wooden Madonnas with tin crowns at every corner; hardware store filled with fragments of old iron, rusty, broken locks &c., over the door carved armorial bearings representing two dragons rampant; bookstore, two or three hundred dirty volumes, with piles of vilely executed prints of Priests, Saints, Madonnas &c.; letter paper with views of Sion *from a distance;* milliner's shop, entered at window, it being the only door, red and yellow ribbons, tawdry bonnets and wooden shoes of all sizes, velvet necklaces with large gold washed clasps to adorn the front of a goitrous tumor; tailor's shop, pictures of Parisian fashions on Parisian dandies at windows, contrasted with a Valais fashion on a Valais tailor at the door; hospital, stone building, refused admission; returning saw two idiots chattering and rocking their senseless heads at each other."

At Sierra, a few miles beyond Sion, conveyances are taken to the warm baths of Leuk, situated five thousand feet up the mountain. The pass is extremely narrow and was defended in 1799 by an inferior force of Vallaisans for several weeks against the French, but the Vallaisans being compelled finally to yield, one fourth of the population of the valley were massacred with nearly all of the cretins, who could neither fly nor defend themselves. The waters of Leuk have a temperature of about thirty-eight degrees, and are much frequented notwithstanding the difficulty of access, and the fact that in 1719 and 1758 every building in Leuk was swept away by avalanches.

Ruined castles have become so familiar to me in this country that I scarcely give them more notice in passing than I do a glacier or a mountain peak; three, stand upon the opposite mountains in sight of Sion, and were once the strong holds of freebooters, who kept the whole adjacent country in awe; above Sierra two more raise their crumbling battlements on the left.

Along the valley of the Visp which is seen opening from the right, lies the pass of Mount Cervin, an hour's ride within which is situated the hamlet of Grenchen, where Thomas Platter, a physician and celebrated reformer was born. Briegg, where we halted for the night, is situated at the northern extremity of the Simplon pass: and worn out with incessant labor, watchfulness and excitement, continued ever since I had left Genava, I was happy to find a warm fire, an excellent supper and a comfortable bed.

I shall now have done with this valley of disease, this strange pathological collection, when I have added a few reflections. I think no one can travel through this Canton without being convinced that, whatever causes may produce goitre elsewhere, here at least it has its source in poverty, filth, indolence, a meagre, coarse diet, humid air and exclusion from light; causes, which from the peculiar disinclination of the inhabitants to migrate or marry beyond their own Canton, have, by ceaseless inheritance been accumulating in intensity through a long succession of generations; for the children of goitrous parents and grand parents are generally *born*

goitrous, and the intermarriage of persons born goitrous produces a cretin offspring. And we may account for the greater prevalence of goitre here than perhaps in any other part of Europe, from the more perfect combination of the causes enumerated, especially exclusion from pure air and light, the valley being the deepest in Europe, and that portion of it which extends between St. Maurice and St. Martigny being very narrow and extending directly north and south, is visited by the sun only a few hours of the day; while the parallel mountains which verge the river, and the lofty barrier of Dent du Midi and Dent de Morcles on the north, and St. Bernard on the south, effectually cut off the wind from whatever point it may blow. Here then is a basin of stagnant air which has never been stirred since the mountains took their present form, and which must forever remain, despite what art or civilization may do, given up to this horrible disease.

Other causes have been assigned for the existence of goitre and cretinism along this valley; none of them however, will bear examination. The "custom of carrying burdens upon the head" so universal here, is no less universal among the peasantry in nearly all parts of Europe, and if it was adequate to the production of goitre here, it would prove equally adequate elsewhere. The "habitual use of snow water" by the Valaisans cannot be denied, since not only the Rhone itself, but all its small tributaries and even most of the springs are supplied directly by the glaciers which are forever above them. Yet how much this has to do with the production of goitre is understood when it is known that a removal to the summits of the mountains where snow water *must* be the sole drink, is the most certain cure of both goitre and cretinism and that if the transplantation occur sufficiently early it will often effectually prevent their development. Nor can I ascribe any more weight to the large amount of "calcareous, magnesian and silicious particles" which those who drink the waters of the Rhone necessarily receive, and which constantly render the river turbid and cover its banks with a white sand, since neither lime, magnesia nor silex have yet been shown to produce goitre. I know many districts in which lime is the only rock and the waters are highly impregnated and yet the malady in question does not prevail—the same it is not denied, may be said of many primitive regions—I attach also great importance to the argument that if foreign particles suspended in the water was actually the cause, it would be so easily ascertained, that it could not fail of having been known long since, by confining, either accidentally or intentionally, persons still residing in the valley to snow artificially melted and deprived of all foreign elements. The proof would be so natural and easy that, I say, it *must* have been made and the doubt resolved.

You remember my suddenly formed opinion, when I saw both goitre and scrofula at Geneva, that both sprang from the same or similar sources, which opinion I declared I would hold with the privilege of changing if I found occasion hereafter. The opinion is confirmed. I do not say positively that the diseases are the same, but certainly they have like causes and many other points in common. Scrofula is hereditary, so is goitre; goitre occurs oftenest in children of light complexion, flaxen hair, large blue eyes, soft, relaxed fibres, and who present not unfrequently a precocity of intellect; the same is true of scrofula. Valaisans have what we term a scrofulous look. Many are rickety or otherwise deformed, yet seldom did I see scrofulous enlargement in the absorbent glands of the neck, and as I believe, because the existence of a tumor often as large as the head and sometimes twice as large, (Dr. Mott says he saw at Martigny one of such magnitude that "the poor woman was obliged to crawl along the floor upon her hands and feet dragging the gigantic dewlap, and pendulous mass after her,") serves as a sufficient drain and prevents their developement—precisely as the existence of one large scrofulous tumor retards the growth of others, while its removal is followed often by a numerous crop of new sprouts along the whole chain of absorbents. I am aware that *in Scotland scrofula is common and goitre is rare*, but having travelled some among the peasantry of the Highlands and the adjacent valleys, and observed their habits, modes of living, climate, I am convinced that to say the least, we are not entitled to the inference drawn by Dr. Gibson that *therefore*, scrofula and goitre cannot be confounded.—Precisely the causes which produce goitre in Switzerland, fair complexion, filthy cabins, meagre, coarse vegetable diet, exclusion from air and light exist also in the valleys of the Scotch Highlands only in a less degree of intensity—and the only inference which could be legitimately made would be that *these causes operating moderately produce scrofula, and operating more intensely produce goitre.*

Farther, the diseases might seem to have a common source, since they are subject to common remedies. Not to be more particular, I may mention the use of iodine, which is regarded as almost equally a specific for both,—tonics,cleanlinesss,nutritious and wholesome diet,and also removal into a more clear and invigorating air. I might also add that while exclusion from light alone has been proven by experiment to produce in dogs "softening of the bones and rickets" (a scrofulous constitution) so here the goitre extends to dogs, goats, sheep, cattle, horses &c.

